# An Interactive Parent-Targeted Text Messaging Intervention to Improve Oral Health in Children Attending Urban Pediatric Clinics: Feasibility Randomized Controlled Trial

**DOI:** 10.2196/14247

**Published:** 2019-11-11

**Authors:** Belinda Borrelli, Michelle Henshaw, Romano Endrighi, William G Adams, Timothy Heeren, Rochelle K Rosen, Beth Bock, Scott Werntz

**Affiliations:** 1 Center for Behavioral Science Research Boston University Henry M Goldman School of Dental Medicine Boston, MA United States; 2 Center for Research to Evaluate & Eliminate Dental Disparities Department of Health Policy & Health Services Research Boston University Henry M Goldman School of Dental Medicine Boston, MA United States; 3 Office of Global & Population Health Boston University Henry M Goldman School of Dental Medicine Boston, MA United States; 4 Department of Pediatrics Boston Medical Center & Boston University School of Medicine Boston, MA United States; 5 Department of Biostatistics Boston University School of Public Health Boston, MA United States; 6 Centers for Behavioral and Preventive Medicine The Miriam Hospital Providence, RI United States; 7 Department of Behavioral and Social Sciences Brown University School of Public Health Providence, RI United States; 8 Department of Psychiatry and Human Behavior Alpert Medical School at Brown University Providence, RI United States; 9 Agile Health, Inc Lincolnshire, IL United States

**Keywords:** oral health, mHealth, text message, dental caries, health behavior

## Abstract

**Background:**

Effective preventive treatments for dental decay exist, but caries experience among preschoolers has not changed, with marked disparities in untreated decay. Despite near-universal use of SMS text messaging, there are no studies using text messages to improve the oral health of vulnerable children.

**Objective:**

This randomized controlled feasibility trial aimed to test the effects of oral health text messages (OHT) versus a control (child wellness text messages or CWT). OHT was hypothesized to outperform CWT on improving pediatric oral health behaviors and parent attitudes.

**Methods:**

Parents with a child aged <7 years were recruited at urban clinics during pediatric appointments (79% [41/52] below poverty line; 66% [36/55] black) and randomized to OHT (text messages on brushing, dental visits, bottle and sippy cups, healthy eating and sugary beverages, and fluoride) or CWT (text messages on reading, safety, physical activity and development, secondhand smoke, and stress) groups. Automated text messages based on Social Cognitive Theory were sent twice each day for 8-weeks. Groups were equivalent on the basis of the number of text messages sent, personalization, interactivity, and opportunity to earn electronic badges and unlock animated characters. Assessments were conducted at baseline and 8 weeks later. Data were analyzed with linear mixed–effects models.

**Results:**

A total of 55 participants were randomized (28 OHT and 27 CWT). Only one participant dropped out during the text message program and 47 (24 OHT and 23 CWT) completed follow up surveys. Response rates exceeded 68.78% (1040/1512) and overall program satisfaction was high (OHT mean 6.3; CWT mean 6.2; 1-7 scale range). Of the OHT group participants, 84% (21/25) would recommend the program to others. Overall program likeability scores were high (OHT mean 5.90; CWT mean 6.0; 1-7 scale range). Participants reported high perceived impact of the OHT program on brushing their child’s teeth, motivation to address their child's oral health, and knowledge of their child's oral health needs (mean 4.7, 4.6, and 4.6, respectively; 1-5 scale range). At follow up, compared with CWT, OHT group participants were more likely to brush their children’s teeth twice per day (odds ratio [OR] 1.37, 95% CI 0.28-6.50) and demonstrated improved attitudes regarding the use of fluoride (OR 3.82, 95% CI 0.9-16.8) and toward getting regular dental checkups for their child (OR 4.68, 95% CI 0.24-91.4). There were modest, but not significant, changes in motivation (F1,53=0.60; *P*=.45) and self–efficacy (F1,53=0.24; *P*=.63) to engage in oral health behaviors, favoring OHT (*d*=0.28 and *d*=0.16 for motivation and self–efficacy, respectively).

**Conclusions:**

The OHT program demonstrated feasibility was well utilized and appealing to the target population and showed promise for efficacy.

## Introduction

### Background

Although there are effective preventive treatments for dental decay, caries experience among preschoolers has remained relatively unchanged for the past two decades [1]. However, not all populations share the burden of the disease equally, demonstrated by the persistent and marked disparities in caries experience, untreated decay, and the lack of dental care access by both race or ethnicity and income [1]. Finding innovative strategies to reduce the prevalence and severity of this disease in high–risk populations is essential to reducing disparities. For young children, the role of the primary caregiver is especially important in reducing caries risk [2].

Community health centers provide comprehensive and cost–effective primary health care for America's most underserved communities. Nationally, there are almost 1400 community health centers with over 11,000 locations that treat 28 million patients per year, of which 8.4 million are children [3]. Community health centers provide care primarily to low–income persons (91%), the under- or uninsured (49% Medicaid; government–funded medical and dental insurance for low–income individuals; and 23% uninsured), and racial and ethnic minority groups (63%) [3]—the same groups that are at highest risk for caries. Despite guidelines that the first dental visit should occur by age 1 year, less than 2% of children enrolled in Medicaid meet this recommendation, and only 9% of 1- and 2-year olds receive preventive dental visits [4]. In contrast, almost 90% of US children attend well–child primary care visits [5]. This high attendance rate, coupled with the fact that during the first 3 years of life, children have 12 well–child visits scheduled, provides an infrastructure with the potential to reach both children at high risk for caries and their parents.

Short message service (SMS) text messaging may be one way to reach busy parents in their everyday lives with evidenced–based information. Over 95% of adults in the United States regularly use SMS text messaging, with no disparities in race, ethnicity, and income [6,7]. The advantages of an SMS text messaging intervention are as follows: access anytime and place, ability to tailor to content timing and intensity, provision of real–time coping strategies to users in everyday settings, few barriers to participation, interactive functionality in real time, low participant burden, reduced cost burden on the health care system [8,9], and high potential for dissemination.

SMS text messaging interventions have been effective across a wide variety of behaviors, such as smoking cessation [10], medication adherence, diabetes care [11], and weight management [12,13]. Although there are 3 studies that use SMS text messaging to improve pediatric oral health, they have small samples and short–term outcomes (1 week-3 months) and lack rigorous controls [14-16]. Only 1 was conducted in the United States and was limited in that it used text messages as *reminders* only, and text messages were sent for only 7 days [14].

We developed a text message program focused on motivating adherence to pediatric oral health behaviors. The program content and structure was based on clinical guidelines [17,18] and recommendations from a multidisciplinary scientific advisory board [19]. We also interviewed medical assistants, nurses, and pediatricians (n=9) to assess their opinions about the program and on how to integrate it into the clinic flow. We conducted 11 focus groups with parents (n=63) to develop text message content, match content to participants’ literacy levels, design program preferences (ie, features, structure, length, and badges), incorporate cultural considerations, identify knowledge gaps, and map text message content onto a theoretical model and mediators (Social Cognitive Theory) [20-22]. To ensure functionality, we conducted an internal pilot (9 users) followed by a usability study during which participants from the target population (n=21) used the text message program for one month and completed self–report questionnaires and qualitative interviews at the middle and end of the month regarding their opinions on program content, badges, and structure as well as satisfaction, comprehension, and perceived impact on hypothesized mediators and behavior.

### Objective

In this paper, we report the results from the text message program that we developed using the iterative process outlined above. Parents who attended the target pediatric clinics and have children under the age of 7 years were randomized into the pilot feasibility randomized controlled trial employing a parallel design and using a 1:1 allocation ratio to receive the oral health text messages (OHT) or child wellness text messages (CWT) for 2 months. Our comparison group, CWT, was developed by our team and scientific advisory board, using clinical guidelines [23,24]. The aim of the pilot was to test recruitment processes and assess participant satisfaction and the potential impact of OHT on putative Social Cognitive Theory mediators, oral health knowledge and attitudes, and pediatric oral health behaviors. We hypothesized that participant satisfaction would be equivalent between conditions (to preserve internal validity), and participants randomized to OHT group would experience positive changes in relevant Social Cognitive Theory constructs (eg, motivation, self–efficacy, and outcome expectations) and report improved knowledge, attitudes, and oral health behaviors (tooth brushing).

## Methods

### Participants

Potential participants were parents (or caregivers) of children who were patients of pediatric clinics in 2 community health centers in an urban area of Boston, United States. The majority of patients in these clinics receive Medicaid (>88%). Participants were recruited to participate in our study, described as a *child wellness study*, by research assistants in pediatric waiting rooms and clinic staff referral. The research assistants administered informed consent to potential participants, and those who consented documented the consent in writing and were asked to complete baseline questionnaires. Participants were then randomized (using random number functions in a 1:1 allocation ratio using the statistical package SAS 9.4 (TS1M5) platform by SAS Institute Inc) to receive either OHT or CWT. Randomization triggered a text message asking the participant to *opt into* the program. A permutated randomized block design was used, stratified by clinic, child age, and history of caries. Research assistants were masked to treatment condition, as participants’ first text messages were delivered 24 hours after enrollment.

Parents were considered eligible if they met the following criteria: aged ≥18 years and had a child aged 6 months to 7 years who received medical care at one of the target community health centers; lived in the greater Boston area and were not planning on moving for 8 weeks; spoke, understood, and read either English or Spanish fluently; had a mobile phone with unlimited SMS text messaging capability; texted at least one time in the past month; adequate ability to read health–related material [25]; were not enrolled in another mobile phone child health or wellness study; reported no abuse of alcohol or drugs [26]; and no previous serious mental illness. The study received ethical approval from our human subject institutional review board along with review and approval by the ethical committees at the community health centers. Participant recruitment and follow up assessment took place from March through May 2017.

### Procedure

#### Structure of the Programs

OHT and CWT were matched on program duration (8 weeks) and dose (2 text messages per day for 1 month followed by 1 text message per day for 1 month), engagement strategies (quizzes on fun facts, birthday text messages, ability to earn child–friendly animated badges for goal achievement, and ability to *unlock* higher levels of animated badges for engaging in the target behavior), and personalization and customization that allowed for the tailoring of message content. Text messages in both conditions were interactive, focusing on problem–solving barriers to behavior change. For OHT, the target goal was brushing every day, twice per day, and for CWT, the target goal was reading every day. Both programs provided feedback on progress toward goal attainment via ability to electronically view a *trophy case* of badges earned so far and motivational text messages. Participants could also participate in *challenge weeks* during which they were given daily electronic badges for achieving the target behavior. The text messages were fully automated, but all incoming text messages could be monitored and responded to in real time via a dashboard interface. Responding to participants directly was necessary if the system did not recognize a text and, therefore, could not produce an automated response—for example, some participants sent a *smiley* emoji or asked a study–related question. The dashboard interface also allowed the study team to respond to participants who did not respond to assessments and allowed for rapid adjustment of personalization settings, such as changing the language of the text messages (ie, English to Spanish or Spanish to English). The dashboard was not used to communicate additional intervention content to participants. Text messages were delivered by Agile Health, Inc. Their system is Health Insurance Portability and Accountability Act compliant, and all data are encrypted in transit and at rest.

#### Oral Health Text Messages

OHT received core topic text messages (tooth brushing and cleaning gums and visiting the dentist) and choice topic text messages (bedtime routine, bottle and sippy cup use, sugar–sweetened beverages, healthy eating, getting fluoride, and fun facts). In month 1, participants received 1 text message from the core topics and 1 text message from the choice topics each day; in month 2, only 1 text message was sent per day, alternating between core and choice topics. Participants earned weekly badges depicting colorful dental–related images if they met the goal of brushing twice each day, working toward the goal of earning a *SuperTooth Hero* badge at the end of each of the 2 months (*Charlie Chew* and *Molly Molar*). Participants could also opt into a *challenge week* in which they were cued daily to brush their child's teeth to unlock *bonus SuperTooth Heroes* (*Faye Fluoride* and *Captain Chomp*) upon achieving brushing twice a day. SuperTooth Heroes were 4 different anthropomorphized teeth wearing capes and holding toothbrushes. The badges and heroes were accompanied by a description of the goal that was achieved. Other text message features specific to OHT included a *dentist finder* (geared to find pediatric dentists), photos and video of brushing technique, and Web links (eg, amount of sugar in popular food and drinks).

#### Child Wellness Text Messages

Participants who were randomized to CWT received core topic text messages focused on the promotion of reading and safety in the home. Choice topic text messages were as follows: healthy sleep and behavior, safety hazards, child development, physical activity, stress tips, and eliminating second hand smoke. CWT earned weekly badges depicting cartoon animals for reading to their child every day. If they earned 4 different weekly badges (each badge specifying the reading goal achieved), they were entitled to *book buddy* badges (anthropomorphized books) each month. They could also earn 2 *mystery book buddies* for the completion of challenge weeks. The badges and buddies were accompanied by a statement of the goal that was achieved. Other text message features specific to CWT included strategies for handling challenging child behaviors, Web links for community resources for parents, and photos or video of important concepts.

### Measures

Surveys were self–administered on the Web at baseline, before randomization, and at the 2-month follow up (after the end of daily text message programs). Participants were compensated a total of US $25 for completing the baseline survey and US $40 for completing the follow up survey. To prevent participant expectations from unduly influencing the results, assessments of CWT (reading and safety) and OHT (oral health behavior) outcomes were given to all participants. As the main purpose of the study was to report on OHT, we have presented the measures and results of only those outcomes.

#### Sociodemographics

Sociodemographics including age, sex, education, income, race and ethnicity, marital and employment status, and child characteristics were obtained through self–report at baseline.

#### Program Satisfaction

Program satisfaction measures were given at the conclusion of the text message programs (2 months after baseline). We used several indices of satisfaction because of the multidimensional nature of program satisfaction. First, we measured the *share–worthiness* of the text messages by asking whether participants showed the text messages to others and the extent to which they believed that the text messages would be helpful to family and friends (ranges from 1=*not at all helpful* to 7=*very helpful*). The perceived quality of the text messages were measured with 2 items from the Mobile Application Rating Scale (MARS) [27]; one assessing perceived quality through a *star* rating (1 star=*one of the worst text message programs*, 3 stars=*average*, and 5 stars=*one of the best text message programs*) and the other assessing how much longer they would have liked to receive the text messages (range from 1=*1 month* to 5=*5 months*). Satisfaction with each program component and overall program satisfaction were assessed with 11 items, each rated on a 1 to 7 scale (range from 1=*not satisfied at all* to 7=*very much satisfied*). The *likeability* of each program component was assessed with 10 items, each rated on a 1 to 7 scale (range from 1=*did not like it at all* to 7=*liked it very much*); we also computed an overall likeability score by averaging across the items. We assessed the perceived impact of the text messages with a 6-item scale from the MARS [27]. This scale assessed the extent to which participants randomized to the OHT group perceived that the program had an impact on their knowledge about oral health, their motivation to brush their child’s teeth, their attitude toward changing their oral health practices, and likelihood of actual behavior change [27]. Each item was rated on a 5-point Likert scale (range from 1=*strongly disagree* to 5=*strongly agree*). We also assessed the perceived impact of the program on 6 key behaviors that corresponded to core and choice topics in the OHT. Participants rated the extent to which the program had an impact on each behavior using a 7-point Likert scale (range from 1=*not at all* to 7=*very much*) [27].

#### Child Brushing and Fluoride Use

Child tooth brushing was assessed as *never*, *sometimes but not every day*, *once a day*, *twice a day*, and *more than twice a day* [28]. Responses were collapsed into 2 levels: yes=achieved brushing recommendations (*twice a day* or *more than twice a day*) versus no (*never*, *sometimes but not every day*, or *once a day*). In the OHT group alone, brushing was assessed weekly through text messages in which participants were asked how many days in the last 7 days were their child’s teeth brushed and how many times each day (1, 2, or more). We computed a brushing behavior variable by multiplying the number of days (0-7) by the number of times per day the child’s teeth were brushed each week. Use of fluoride toothpaste was assessed with 1 item, “when your child's teeth are brushed, is fluoride toothpaste usually used (yes/no)?” [28].

#### Attitudes Toward Oral Health

Attitudes toward oral health were assessed with 3 items from the Basic Risk Factors Questionnaire [29-32]: (1) “Children can get cavities as soon as their first tooth comes in,” (2) “It is best to use toothpaste with fluoride when brushing a child’s teeth,” and (3) “Children’s teeth should be brushed the last thing before bed.” Participants rated each on a 4-point scale (1=*strongly disagree*, 2=*somewhat disagree*, 3=*somewhat agree*, and 4=*strongly agree*). For analyses, *strongly disagree*, *somewhat disagree*, and *somewhat agree* were collapsed into a *not strongly agree* category and compared against the *strongly agree* group.

#### Social Cognitive Theory Constructs

Social Cognitive Theory constructs were assessed with measures from the Basic Risk Factors Questionnaire [32]. Outcome expectations or beliefs that engaging in a behavior will produce a desired outcome was measured with 3 items: (1) “Limiting my child’s intake of sugary foods and drinks can help prevent cavities,” (2) “Drinking tap water can help prevent cavities,” and (3) “Regular dental checkups help keep children’s teeth and mouth healthy,” each rated on a 4 point scale (1=*strongly disagree*, 2=*somewhat disagree*, 3=*somewhat agree*, and 4=*strongly agree*). *Strongly disagree*, *somewhat disagree*, and *somewhat agree* were collapsed into *not strongly agree* and compared against *strongly agree*. Motivation was assessed with 4 items measuring participants’ degree of desire to engage in recommended oral health behaviors, each rated on a scale ranging from 1 to 5 (higher scores reflect higher motivation). Self–efficacy was assessed with 4 items that assessed participants’ perceived degree of confidence in their ability to engage in recommended oral health behaviors, each item rated on a scale ranging from 1 to 5 (higher scores reflect greater self–efficacy).

#### Program Engagement

Engagement was collected automatically through program interaction. We computed *dose received* by dividing the number of text messages sent to participants each week by the number of participants and then averaging across all weeks. A *total response rate* was computed by computing the number of participant–submitted responses to texts in which a response was expected and dividing this by the number of possible responses. An *assessment response rate* was computed by dividing the number of participants that responded to assessment texts by the number of participants. The number of *unsolicited* user texts (texts sent by users where a response was not expected such as *emojis* and *thank you*) was an additional index of user engagement. We also tracked the number of participants who opted into *challenge weeks* in which users were to set a daily behavioral goal (for the OHT group participants, the *challenge* was brushing their child’s teeth twice per day, and for the CWT group participants, the *challenge* was reading to their child for 10 min each day). For OHT group, we also assessed the proportion of participants choosing each *choice* module across the 8-week program. This was computed by combining the number of OHT participants that selected any given *choice* module every week the module was available and dividing it by the number of potential module choices (product of number of weeks a module was made available and sample size).

### Analytic Plan

At baseline, study groups were compared on sociodemographic characteristics using independent sample *t* tests for continuous variables or chi–square tests for categorical variables. The baseline characteristics of participants who did not complete the follow up survey (n=8) were also compared with the rest of the sample using *t* tests or chi–square tests as appropriate. User engagement and interaction with the program data were summarized and compared between study groups. Program satisfaction was compared between groups with *t* tests, and perceived program impact descriptives are presented for the OHT group.

Changes in oral health attitudes and behaviors from baseline to follow up in the OHT group compared with the CWT group were analyzed through models for longitudinal data with a group–by–time interaction representing the intervention effect. For binary outcomes, Generalized Estimating Equations (GEE) logistic regression for longitudinal data estimated the odds of achieving a behavior (eg, brushing recommendations or use of fluoride toothpaste) at follow up compared with baseline, for those in the OHT versus CWT group. For outcome expectations and attitudes toward oral health, GEE logistic regression estimated the odds of strongly versus not strongly agreeing to each construct item at follow up versus baseline, for the OHT versus CWT group. For continuous outcomes, mixed–effects linear regression models were used to compare changes in group means from baseline to follow up, in the OHT versus CWT group. Effect sizes for continuous measures are presented as Cohen *d* calculated on the change score between baseline and follow up. Effect sizes for binary measures are presented as odds ratios (OR) with 95% confidence intervals. Analyses were conducted on participants who completed the follow up assessment (n=47).

## Results

### Overview

**Figure 1:**
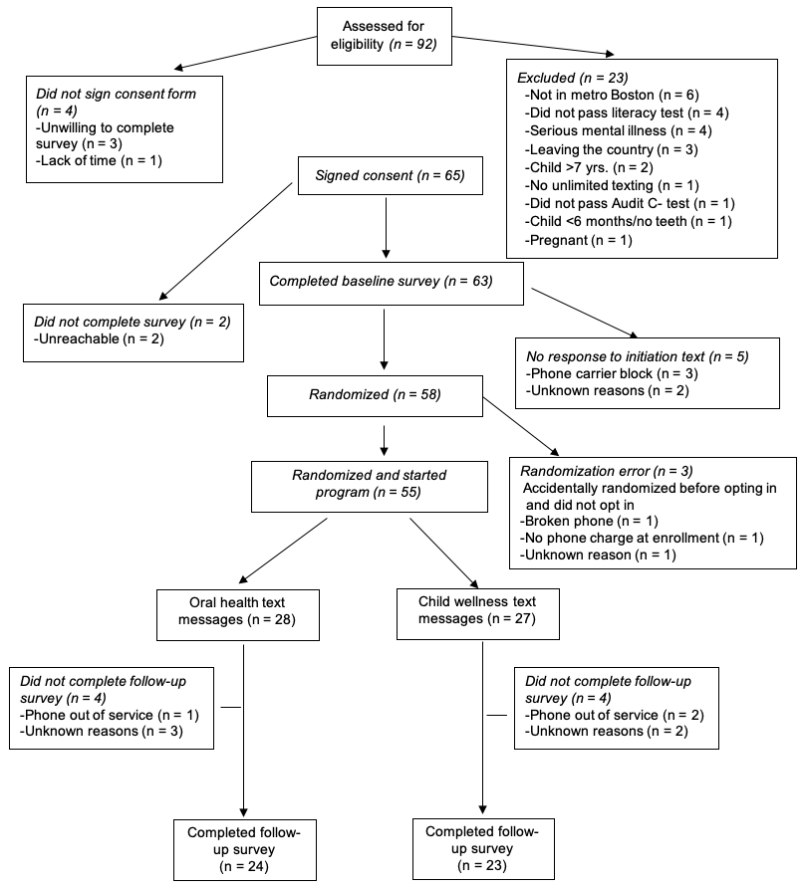
Consolidated Standards of Reporting Trials diagram.

As shown in Figure 1, 55 out of the 65 individuals (55/65, 85%) who were eligible and signed the informed consent were randomized and initiated the text messages programs; 47 out of the 55 randomized participants (47/55, 85%) completed the end of treatment assessment with no significant differences in completion rates between groups. Participants who did not complete the follow up survey (n=8) were significantly younger (mean age in years 26.8 vs 31.6; *P*=.02). Satisfaction data were completed by 48 participants. Only 1 participant dropped out of the text message program. Owing to a technical problem, there were 3 CWT that were scheduled to be delivered but were not delivered. A total of 2 of these text messages applied only to participants who had toddlers, and 1 text message applied only to those who chose the stress management module. Therefore, this technical problem affected <2% of the text messages. There were no other unintended or harmful effect to participants.

### Sociodemographics

Compared with OHT, CWT group participants were more likely to be employed (χ^2^_4_=14.7; *P*=.005) and have received information about children’s dental health at pediatric visits in the past year (χ^2^_1_=4.1; *P*=.04). The mean age of the child was 2.7 years (SD 1.7) in the OHT group and 3.0 years (SD 1.9) in the CWT group. Out of the 28 children, 6 (6/28, 21%) in the OHT group, and out of the 27 children, 5 (5/27, 18%) in the CWT group had a history of cavities, reported via parent self–report. The baseline characteristics of the participants are shown in Table 1.

**Table 1:** Baseline characteristics of participants by treatment group.

Baseline characteristics		Oral health text messages (n=28), n (%)	Child wellness text messages (n=27), n (%)	All (N=55), n (%)
Female		28 (100)	25 (93)	53 (96)
Parent/caregiver age (year), mean (SD)		31.0 (6.4)	31.0 (6.9)	31.0 (6.6)
Below poverty line		23 (88)^a^	18 (69)^a^	41 (79)
Less than high school education		6 (21)	2 (7)	8 (15)
Full/part time employment^b^		8 (29)	21 (78)	29 (53)
Married/engaged/live together		10 (36)	5 (19)	15 (27)
**Race/ethnicity**				
	Black—African American	18 (64)	18 (67)	36 (66)
	White—non-Hispanic	4 (14)	0 (0)	4 (7)
	Asian	2 (7)	1 (4)	3 (5)
	Hispanic (black/white)	3 (11)	6 (22)	9 (16)
	Multiracial/other	1 (4)	2 (7)	3 (6)
Received dental health information^c^		9 (32)	16 (59)	25 (45)
Use fluoride toothpaste		12 (46)^a^	12 (48)^d^	24 (47)
**Mobile communication preference^e^**				
	Text message	21 (78)	21 (78)	42 (78)
	Phone call	2 (7)	2 (7)	4 (7)
	No preference	4 (15)	4 (15)	8 (15)
**Texting frequency^e^**				
	Every day	21 (78)	24 (89)	45 (83)
	Most days	6 (22)	1 (4)	7 (13)
	Occasionally	0 (0)	2 (7)	2 (4)
**Number of text messages sent per day**				
	1-3	4 (14)	2 (7)	6 (11)
	4-10	7 (25)	9 (33)	16 (29)
	≥11	17 (61)	16 (59)	33 (60)

^a^n=26.

^b^*P*=.005.

^c^*P*=.04.

^d^n=25.

^e^Oral health text messages, n=27.

### Program Satisfaction

In the OHT group, 8 out of 25 participants (8/25, 32%) showed the text messages to others compared with 11 out of 23 (11/23, 47%) in the CWT group (χ^2^_1_=1.0; *P*=.32). Participants in both groups believed that the text messages would be helpful to family/friends (1-7 scale; OHT mean 5.7, SD 1.6; CWT mean 6.0, SD 1.0; *t*_47_=−0.9; *P*=.35), and 84% (21/25) of OHT group participants said that they would recommend the program to others. In the OHT group, 15 out of 22 (15/22, 68%) participants rated the program 4 stars or more compared with 16 out of 21 (16/21, 78%) in the CWT group (χ^2^_1_=0.6; *P*=.34). Out of 19 participants, 12 (12/19, 63%) in the OHT group wanted the program to last up to 2 months longer, and 7 participants (7/19, 37%) indicated that they wanted the program to last ≥3 months longer, which was not significantly different from the CWT group (χ^2^_1_=1.8; *P*=.18). Satisfaction ratings were generally high for program components (Table 2). The mean overall level of program satisfaction was also high with no significant group differences (1-7 scale range; OHT mean 6.3, SD 1.4; CWT mean 6.2, SD 0.9; *t*_46_=0.4; *P*=.70).

**Table 2:** Participant's satisfaction with the text message program.

Program satisfaction scale items	Oral health text messages (n=25), mean (SD)	Child wellness text messages (n=23), mean (SD)
The ability to choose topics of interest to me	6.29 (1.4)	6.21 (1.0)
The ability to earn electronic badges	5.66 (1.8)	5.72 (1.2)
The ability to unlock levels of badges	5.63 (1.9)	5.66 (1.3)
The level of program customization to child	6.25 (1.4)	5.78 (1.2)
Receipt of support when needed	6.20 (1.1)	5.65 (1.0)
The amount of information in the text messages	6.24 (1.3)	5.86 (1.3)
The quality of the information in the text messages	6.08 (1.4)	5.86 (1.0)
Relevancy of program was for self and family	6.32 (1.4)	5.95 (1.1)
Age–appropriateness of the messages for child	6.00 (1.4)	6.21 (0.8)
The trustworthiness of the information	6.33 (0.8)	6.17 (0.9)
The degree to which the text messages apply to their family	5.96 (1.6)	6.00 (1.0)

With regard to the *likeability* of program components (Table 3), there were no significant differences between groups in overall *likeability* scores (1-7 scale range; OHT mean 5.90, SD 1.40; CWT mean 6.00, SD 0.80; *t*_46_=−0.4; *P*=.70). Participants in both groups liked the frequency of the text messages (mean 5.75, SD 1.60), timing of the text messages (mean 5.74, SD 1.4) and text message features such as the ability to set goals (mean 6.21, SD 1.00), choose topics of interest (mean 6.40, SD 1.00), earn weekly badges (mean 5.68, SD 1.60), and participate in challenge weeks (mean 6.55, SD 0.70). With regard to the perceived impact of the program, OHT group participants indicated that taking part in the program increased their awareness of their child’s oral health (1-5 scale range; mean 4.64, SD 1.10), increased their knowledge and understanding of their child’s oral health needs (mean 4.64, SD 1.00), increased their motivation to address their child’s oral health (mean 4.64, SD 1.00), improved their attitude toward their child’s oral health (mean 4.64, SD 0.80), encouraged them to bring their child to the dentist for regular checkups (mean 4.48, SD 1.10), and helped them ensure their child’s teeth were brushed (mean 4.68, SD 1.0). OHT group participants also indicated that the program had a positive impact on brushing their child’s teeth (1-7 scale range; mean 6.40, SD 1.50), increasing the amount of their child’s tap water consumption (mean 5.48, SD 2.00), and decreasing their child’s consumption of sugar–sweetened beverages (mean 6.32, SD 1.30) and sugary foods (mean 6.12, SD 1.50). OHT group participants also indicated that the program had a positive impact on their willingness to take their child to the dentist (mean 5.84, SD 2.0) and overall knowledge about their child's oral health (mean 6.20, SD 1.5).

**Table 3:** Likeability of the text message program.

Likeability scale items	Oral health text messages (n=25), mean (SD)	Child wellness text messages (n=23), mean (SD)
Responding to text message questions on a daily basis	5.52 (1.8)	5.82 (1.2)
Responding to text messages about the frequency of the target behavior (brushing or reading)	5.88 (1.6)	6.00 (1.0)
Setting goals	6.41 (0.9)	6.00 (1.2)
The ability to choose text message topics of interest to you	6.25 (1.4)	6.56 (0.6)
Receiving information about a particular topic	6.29 (1.4)	6.30 (0.8)
The frequency with which texts were delivered	5.64 (2.0)	5.86 (1.3)
The time of the day texts were received	5.83 (1.6)	5.65 (1.2)
Earning weekly badges	5.65 (2.0)	5.72 (1.4)
Earning monthly animated characters (*SuperTooth Heroes* or *Book Buddies*)	5.60 (2.0)	5.47 (1.3)
Participating in a *challenge week*	6.79 (0.5)	6.30 (0.8)

### Child Brushing and Fluoride Use

The proportion of participants meeting pediatric brushing recommendations increased from baseline to follow up in both groups, with the effect size favoring OHT (OR 1.37, 95% CI 0.28-6.50). In the OHT group, brushing was also assessed weekly through text messages, and increased rates of brushing was reported over the course of the 8-week program (time effect *F*_1,94_=8.4; *P*=.005; see Multimedia Appendix 1). The proportions of participants using fluoride toothpaste to brush their child’s teeth increased from baseline to follow up in both groups, with the effect size favoring CWT (OR 0.81, 95% CI 0.25-2.65).

### Attitudes Toward Oral Health

As shown in Table 4, the proportion of participants strongly agreeing that “children can get cavities in baby’s teeth” increased from baseline to follow up in both groups, with the effect size favoring the OHT group (OR 1.98, 95% CI 0.54-7.18). The proportion of participants strongly agreeing that “it is best to use toothpaste with fluoride when brushing a child’s teeth” increased from baseline to follow up in the OHT group alone (OR 3.82, 95% CI 0.90-16.80), and the proportion strongly agreeing that “children’s teeth should be brushed the last thing before bed” increased in both groups, with the effect size favoring the OHT group (OR 2.07, 95% CI 0.10-41.50).

**Table 4:** Oral health behaviors and attitudes.

Oral health behavior and attitudes		Baseline		Follow up		Odds Ratio (95% CI)
		OHT^a^ n/N, n (%)	CWT^b^ n/N, n (%)	OHT n/N, n (%)	CWT n/N, n (%)	
**Oral health behavior**						
	Achieved child brushing recommendations^c^	17/26 (65)	15/24 (62)	17/22 (77)	16/23 (70)	1.37 (0.28-6.5)
	Used fluoride toothpaste to brush child’s teeth	12/26 (46)	12/25 (48)	14/22 (64)	14/21 (67)	0.81 (0.25-2.65)
**Attitudes toward oral health (strongly agree)**						
	Children can get cavities in baby's teeth	6/25 (24)	10/26 (38)	11/24 (46)	11/22 (50)	1.98 (0.54-7.18)
	Best to use fluoride toothpaste for children	9/21 (43)	15/24 (62)	15/22 (68)	12/22 (54)	3.82 (0.90-16.8)
	Should brush teeth last thing before bed	21/25 (84)	22/26 (85)	23/24 (96)	21/23 (91)	2.07 (0.10-41.50)
**Outcome expectations (strongly agree)**						
	Limiting sugary foods helps prevent cavities	19/25 (76)	19/26 (73)	21/24 (87)	18/23 (78)	1.68 (0.23-11.80)
	Drinking tap water helps prevent cavities	5/22 (23)	4/26 (15)	8/22 (36)	4/20 (20)	1.42 (0.24-8.60)
	Dental checkups help keep teeth healthy	20/26 (77)	23/26 (88)	23/24 (96)	21/23 (91)	4.70 (0.24-91.40)

^a^OHT: oral health text message.

^b^CWT: child wellness text message.

**^c^**Brushing twice per day, 6 or more days per week.

### Social Cognitive Theory Constructs

With regard to outcome expectations, Table 4 shows that the proportion of participants strongly agreeing that “limiting children’s intake of sugary foods helps prevent cavities” increased from baseline to follow up in both groups, with the effect size favoring the OHT group (OR 1.68, 95% CI 0.23-11.80). There was a similar pattern for the belief that “drinking tap water can help prevent cavities,” with the effect size favoring the OHT group (OR 1.42, 95% CI 0.24-8.60), and for “regular dental check–ups help keep children’s teeth and mouth healthy,” with the effect size in favor of the OHT group (OR 4.68, 95% CI 0.24-91.40).

Participants' motivation to engage in oral health promoting behaviors score increased between baseline (OHT mean 4.31 and CWT mean 4.42) and follow up (OHT mean 4.64 and CWT mean 4.52) in both groups, with the effect size favoring the OHT group (d=0.28) and no significant between–group differences (*F*_1,53_=0.60; *P*=.45). A similar pattern emerged for self–efficacy. Scores increased between baseline (OHT mean 4.50 and CWT mean 4.60) and follow up (OHT mean 4.76 and CWT mean 4.76) in both groups, with the effect size favoring the OHT group (d=0.16) and no significant between–group differences (*F*_1,53_=0.24; *P*=.63).

### Program Engagement

The mean weekly number of texts sent was comparable between groups (15.3 in OHT vs 15.4 in CWT), indicating dose equivalence. Aggregating over the 8-week study period, in the OHT group, there were 1040 responses out of the 1512 (1040/1512, 68.8% overall total response rate) possible responses, whereas in the CWT group, there were 1167 responses out of the 1463 (1167/1463, 79.8% overall total response rate) possible responses. Response to text messages did not taper off as the program progressed, remaining consistent across weeks for both groups (see Multimedia Appendix 2). Averaging across the study period, of the 28 OHT group participants, the mean number of participants responding to weekly assessment texts was 19.3 (19.3/28, 69% overall assessment response rate). Of the 27 CWT group participants, the mean number of participants responding to weekly assessment texts was 19 (19/27, 70% overall assessment response rate). Of the 28 OHT group participants, 16 (16/28, 57%) opted into at least one of the 2 possible *challenge weeks*. Of the 27 CWT group participants, 17 (17/27, 63%) opted into at least one of the 2 possible *challenge weeks*. Participants in the OHT group sent a total of 439 *unsolicited* text messages and participants in the CWT group sent a total of 475 unsolicited text messages during the 8 week program. With regard to choosing *choice* topics, aggregating over 8 weeks when *bedtime routine* was offered, 42% (35/84) selected the module; when *bottle/sippy cup* was offered, 21% (23/112) selected the module; when *fun facts* was offered, 30% (25/84) selected the module; when *getting fluoride* was offered, 32% (27/84) selected the module; when *healthy eating* was offered, 51% (57/112) selected the module; and when *sugar–sweetened beverages* was offered, 26% (29/112) selected the module.

## Discussion

### Principal Findings

Sustainability of health behavior change is greatest if interventions are integrated into existing channels and woven into the fabric of people's lives. Our study incorporated both of these elements by partnering with pediatric clinics regularly visited by underserved families and using text messages, a preferred and near–universal form of communication. Text message interventions have the advantage of reaching large segments of previously unreachable populations with evidenced–based information, in real time and real–life settings. No previous studies have tested text messages to improve the oral health of at–risk children in a randomized controlled trial, matching for treatment dose and intensity. Our pilot study showed proof of concept of our OHT intervention with 4 principal findings: (1) OHT was perceived as highly acceptable and satisfactory, (2) participants in both conditions demonstrated a high level of engagement, (3) OHT had an impact on parent’s attitudes toward oral health and social cognitive mediators, and (4) the program showed preliminary effectiveness at increasing brushing behaviors among those randomized to the OHT versus CWT group.

The high levels of acceptability and satisfaction reported by participants could be a function of the fact that we co–designed the program content and structure with the target population. Focus groups and interviews enabled us to ascertain participant preferences about the *surface structure* of the program (look and feel and images of the electronic badges) and the *deep structure* of the program (values and beliefs of the population) [33]. Collecting both quantitative and qualitative data during program development helped ensure that the words and images used were acceptable and incorporated cultural preferences and ensure that we could address any knowledge gaps and myths about oral health behaviors. We assessed satisfaction not only for the program as a whole but also for each program component, which has been rarely reported in the literature but is essential for the design of effective programs. Aside from self–report of satisfaction, another indicator of program satisfaction is whether or not participants report sharing the text messages with others. A large minority of our participants indicated that they shared the texts with family and friends. Thus, the program could also have a *contagion* effect, that is, unmeasured effects within each person’s social network.

Our strategies for engagement not only included *static* strategies, such as personalization and customization, but also included *dynamic* strategies that required participant interaction, such as quizzes on fun facts, the ability to earn child–friendly electronic badges, and enticement to *unlock* access to other characters. Program engagement was high on all 3 indicators and did not significantly differ between groups. It is important to have several indicators of engagement to avoid masking differential engagement rates, such as those that might exist between overall program responses and responses to research–related text messages. In addition, few, if any studies, have reported on *unsolicited* texts from participants, but this is also an important indicator of engagement because proactive responding could be an indicator of deeper processing of the text messages by participants, rather than simply reacting to text messages. Of the 3 studies that have used text messages for pediatric oral health, only 1 reported on program engagement, but that program was only 7 days in duration [14].

Few studies have examined the effect of a pediatric oral health intervention on parental attitudes, and previous studies have supported the association between attitudes and behavior [28]. Our findings indicated that, with the exception of using fluoridated toothpaste to brush their child’s teeth, OHT consistently showed promise for changing caregiver attitudes and behaviors toward their child’s oral health. No studies to date have used theory (Social Cognitive Theory) to develop a comprehensive oral health intervention to improve children’s oral health through text messages. It may be that the development of text messages grounded in theory is an important factor in improving both parental attitudes and behaviors toward child’s oral health. This is supported by the fact that OHT also showed promise for changing variables integral to the Social Cognitive Theory, such as motivation, self–efficacy, and outcome expectations.

Feasibility studies are used to determine whether an intervention is appropriate for further testing, particularly when there are few or no published studies on a particular intervention technique [34]. Our study met the criteria for intervention feasibility outlined by Bowen et al [34]. Specifically, we demonstrated acceptability (satisfaction and perceived appropriateness), demand (participants were engaged with the program), implementation (successful execution; no technical problems with the text messages), practicality (ease and quality of implementation and low burden on patients and providers), integration (fit into clinic work flow and lack of disruption of clinical care), and limited efficacy (intended effects of the program on key variables; perceived impact).

### Limitations

The primary purpose of the study was feasibility rather than a fully powered clinical trial, so caution should be used when interpreting group differences because of the lack of power. The small sample precludes generalization to the larger population, and as the sample was mostly women and those whose income was below the poverty line, it is unclear if the program would also be acceptable to men and those having higher incomes. Generalizability was also limited by our inclusion and exclusion criteria, which included adequate ability to read health–related material, no previous or current serious mental illness, and no current alcohol or drug abuse. These limitations are offset by the strengths of our study design, which matched groups on text message dose, frequency, and features; creative engagement strategies; objective measurement of engagement; targeting a high–risk population; and implementation in a real–world setting.

### Conclusions

Dissemination of text message interventions is highly viable given the high rate of SMS text messaging and lack of disparities by income, race, or ethnicity. Text message interventions could be disseminated at low cost and are delivered exactly as designed, resulting in 100% reliable intervention. This study provides evidence that a larger fully powered randomized controlled trial with objective outcomes (clinical exam) should be conducted. If effective, the program could be disseminated nationally to other federally qualified pediatric clinics that serve vulnerable and high–risk populations.

## Appendix

Multimedia Appendix 1 Engagement and interaction with the text message program over time.

Multimedia Appendix 2 Changes in child brushing over time in the oral health text messages group.

Multimedia Appendix 3 CONSORT-EHEALTH checklist (V 1.6.1).

## Abbreviations

CWT: child wellness text messages
GEE: Generalized Estimating Equations
MARS: Mobile Application Rating Scale
OHT: oral health text messages
OR: odds ratio
SMS: short message service
