# Supplementary material for: An Interactive Parent-Targeted Text Messaging Intervention to Improve Oral Health in Children Attending Urban Pediatric Clinics: Feasibility Randomized Controlled Trial
Source: JMIR Mhealth Uhealth. 2019 Nov 11;7(11):e14247. doi: 10.2196/14247 (PMC6878100; doi:10.2196/14247)
Supplement: Multimedia Appendix 1 [file mhealth_v7i11e14247_app1.pdf]

Multimedia Appendix 1. Engagement and interaction with the text message program over time.

	**OHT**			**CWT**		
	Total Response Rate	Assessment Response Rate n=28	User Interaction	Total Response Rate	Assessment Response Rate n=27	User Interaction
**Time**						
Week-1	93/138 (67.4%)	82%	81	98/142 (69.0%)	78%	95
Week-2	145/191 (75.9%)	71%	64	107/120 (89.2%)	70%	76
Week-3	128/193 (66.3%)	64%	59	90/113 (79.6%)	74%	56
Week-4	131/184 (71.2%)	68%	40	102/137 (74.5%)	78%	62
Week-5	153/272 (56.3%)	71%	58	258/323 (79.9%)	74%	75
Week-6	114/158 (72.2%)	64%	50	148/192 (77.1%)	63%	39
Week-7	136/207 (65.7%)	57%	52	177/229 (77.3%)	63%	39
Week-8	140/169 (82.8%)	68%	35	187/207 (90.3%)	74%	33

Note. OHT = oral health texts, CWT = child wellness texts. Total Response Rate = number of participants' responses/number of expected responses; Assessment Response Rate = number of participants responding to assessment texts/number of participants; User Interaction = number of unsolicited texts sent by participants (see Methods for details).
