# Supplementary material for: An Interactive Parent-Targeted Text Messaging Intervention to Improve Oral Health in Children Attending Urban Pediatric Clinics: Feasibility Randomized Controlled Trial
Source: JMIR Mhealth Uhealth. 2019 Nov 11;7(11):e14247. doi: 10.2196/14247 (PMC6878100; doi:10.2196/14247)
Supplement: Multimedia Appendix 2 [file mhealth_v7i11e14247_app2.pdf]

Multimedia Appendix 2. Changes in child brushing over time in the OHT group

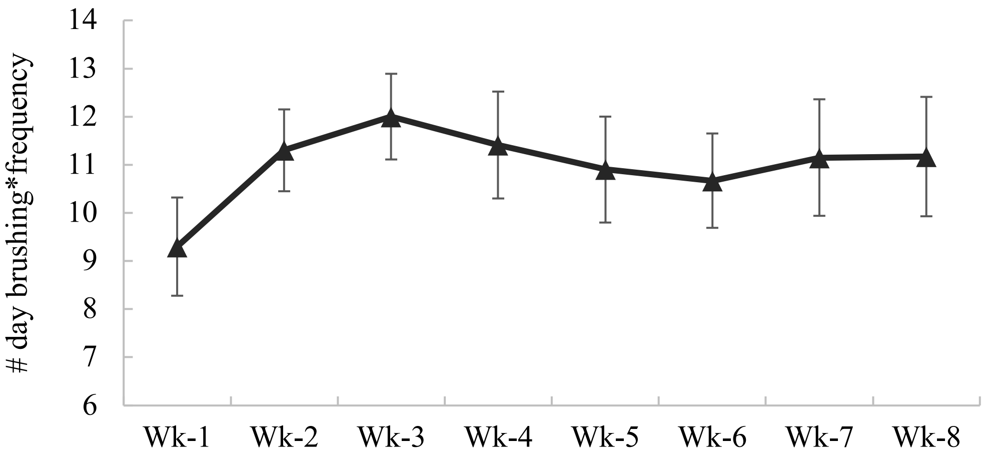

Note. OHT = oral health texts, Wk = week. Wk-1 n = 22, Wk-2 n = 18, Wk-3 n = 18, Wk-4 n = 19, Wk-5 n = 20, Wk-6 n = 18, Wk-7 n = 16, Wk-8 n = 19.
